# (*E*)-1-[(2-Hy­droxy-1-naphth­yl)methyl­idene­amino]­imidazolidine-2,4-dione

**DOI:** 10.1107/S1600536810020118

**Published:** 2010-06-09

**Authors:** Liang-Quan Sheng, Hua-Jie Xu, Na-Na Du, Xue-Yue Jiang

**Affiliations:** a Department of Chemistry, Fuyang Normal College, Fuyang Anhui 236041, People’s Republic of China

## Abstract

The title compound, C_14_H_11_N_3_O_3_, adopts an *E* or *trans* configuration with respect to the C=N bond. In the mol­ecule there is an intra­molecular O—H⋯N hydrogen bond involving the hy­droxy substituent at the 2-positon of the naphthalene ring and the adjacent methyl­ene­amino N atom. The mol­ecule is roughly planar, the dihedral angle between the naphthalene and imidazolidine-2,4-dione mean planes being 8.4 (1)°. In the crystal, pairs of N—H⋯O hydrogen bonds link mol­ecules into inversion dimers. These dimers are futher linked *via* C—H⋯O inter­actions, forming a three-dimensional network.

## Related literature

For the naphthalene group as a fluoro­phore, see: Li *et al.* (2010 ▶); Iijima *et al.* (2010 ▶). For a related structure, see: Xu *et al.* (2009 ▶). For bond-length data, see: Allen *et al.* (1987 ▶).
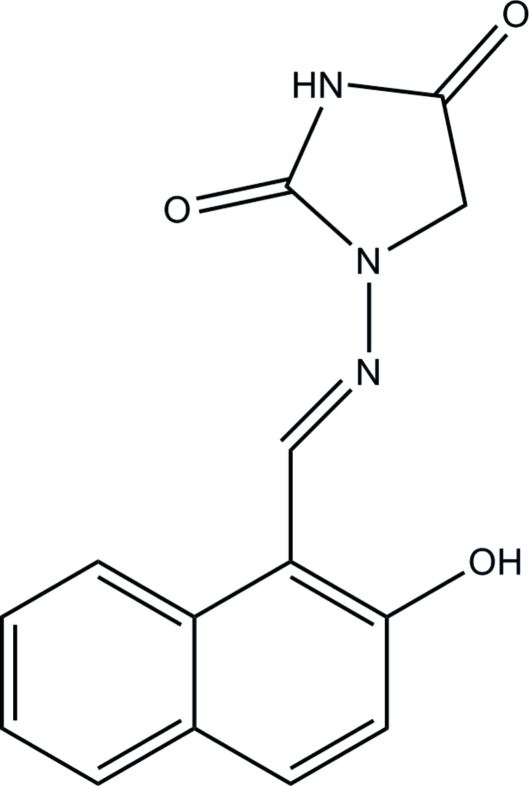


## Experimental

### 

#### Crystal data

C_14_H_11_N_3_O_3_
*M* *_r_* = 269.26
Monoclinic, 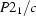

*a* = 11.5122 (7) Å
*b* = 6.0233 (3) Å
*c* = 17.9955 (10) Å
β = 96.773 (5)°
*V* = 1239.13 (12) Å^3^
*Z* = 4
Mo *K*α radiation
μ = 0.11 mm^−1^
*T* = 293 K
0.40 × 0.30 × 0.30 mm

#### Data collection

Oxford Diffraction Gemini S Ultra diffractometer
Absorption correction: multi-scan (*CrysAlis PRO*; Oxford Diffraction, 2009 ▶) *T*_min_ = 0.959, *T*_max_ = 0.969
4288 measured reflections
2136 independent reflections
1053 reflections with *I* > 2σ(*I*)
*R*_int_ = 0.031

#### Refinement

*R*[*F* ^2^ > 2σ(*F* ^2^)] = 0.036
*wR*(*F* ^2^) = 0.067
*S* = 0.72
2136 reflections
186 parameters
H atoms treated by a mixture of independent and constrained refinement
Δρ_max_ = 0.11 e Å^−3^
Δρ_min_ = −0.13 e Å^−3^

### 

Data collection: *CrysAlis PRO* (Oxford Diffraction, 2009 ▶); cell refinement: *CrysAlis PRO*; data reduction: *CrysAlis PRO*; program(s) used to solve structure: *SHELXS97* (Sheldrick, 2008 ▶); program(s) used to refine structure: *SHELXL97* (Sheldrick, 2008 ▶); molecular graphics: *SHELXTL* (Sheldrick, 2008 ▶); software used to prepare material for publication: *SHELXL97*.

## Supplementary Material

Crystal structure: contains datablocks global, I. DOI: 10.1107/S1600536810020118/su2180sup1.cif

Structure factors: contains datablocks I. DOI: 10.1107/S1600536810020118/su2180Isup2.hkl

Additional supplementary materials:  crystallographic information; 3D view; checkCIF report

## Figures and Tables

**Table 1:** Hydrogen-bond geometry (Å, °)

*D*—H⋯*A*	*D*—H	H⋯*A*	*D*⋯*A*	*D*—H⋯*A*
N3—H3*N*⋯O1^i^	0.890 (18)	1.962 (18)	2.851 (2)	177.9 (19)
O3—H4⋯N1	0.82	1.91	2.622 (2)	145
C6—H6⋯O2^ii^	0.93	2.55	3.469 (2)	169
C14—H14*B*⋯O2^ii^	0.97	2.36	3.038 (2)	127

Symmetry codes: (i) 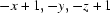; (ii) 
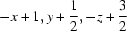
.

**Fig. 1.**
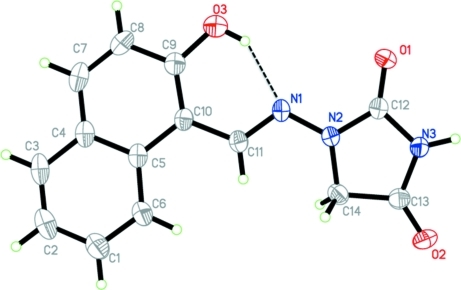
The molecular structure of the title compound, showing 30% probability displacement ellipsoids. The intramolecular O-H···N hydrogen bond is shown as a dashed line (see Table 1 for details).

**Fig. 2.**
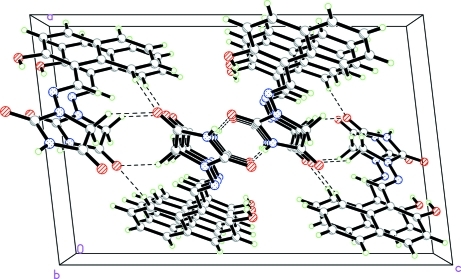
Crystal packing viewed along the b-axis of the title compound. The intra- and intermolecular hydrogen bonds are shown as dashed lines (see Table 1 for details).

## supplementary crystallographic information

### Comment

The naphthalene group as a fluorophore has been studied extensively due to its characteristic photophysical properties and the competitive stability in the environment (Li *et al.*, 2010; Iijima *et al.*, 2010). As part of an ongoing study of Schiff bases incorporating the naphthalene group (Xu *et al.*, 2009), we report here on the crystal structure of the title compound.

The molecular structure of the title compound is shown in Fig. 1. It can be seen to display a *trans* configuration about the C\bN bond. The bond lengths are normal (Allen *et al.*,  1987). There is an intramolecular O-H···N hydrogen bond (Table 1) and the molecule is relatively planar; dihedral angle involving the naphthalene mean plane and the imidazolidine-2,4-dione group mean plane being 8.4 (1)°. In the crystal structure of the title compound N—H···O intermolecular hydrongen bonds (Table 1) link two molecules to form dimmers situated about an inversion center. The molecules are further linked by weak C-H···O interactions to form a three-dimensional network (Fig. 2).

### Experimental

The solution of 1-Aminohydantoin hydrochloride (0.151 g, 1 mmol) in 5 ml of ethanol was added slowly to a solution containing 2-hydro-1- naphthaldehyde (0.172 g, 1 mmol) in 15 ml of absolute ethanol under heating and stirring. The mixture was then refluxed for 2 h. Afterwards the mixture was cooled to rt and the resulting solution to stand in air. After 15 days yellow block-shaped crystals were formed, on slow evaporation of the solvent.

### Refinement

The N3 H-atom was located in a difference Fourier map and freely refined: N-H = 0.89 (2) Å. All other H-atoms were placed in calculated positions and treated as riding: O—H = 0.82 Å, C—H = 0.93 or 0.97 Å, with *U*_iso_(H) = 1.5 *U*_eq_(parent O-atom) and = 1.2*U*_eq_(parent C-atom).

### Crystal data

**Table:**

C_14_H_11_N_3_O_3_	*F*(000) = 560
*M**_r_* = 269.26	*D*_x_ = 1.443 Mg m^−^^3^
Monoclinic, *P*2_1_/*c*	Mo *K*α radiation, λ = 0.71073 Å
Hall symbol: -P 2ybc	Cell parameters from 1596 reflections
*a* = 11.5122 (7) Å	θ = 3.1–29.0°
*b* = 6.0233 (3) Å	µ = 0.11 mm^−^^1^
*c* = 17.9955 (10) Å	*T* = 293 K
β = 96.773 (5)°	Block, yellow
*V* = 1239.13 (12) Å^3^	0.40 × 0.30 × 0.30 mm
*Z* = 4	

### Data collection

**Table:**

Oxford Diffraction Gemini S Ultra diffractometer	2136 independent reflections
Radiation source: fine-focus sealed tube	1053 reflections with *I* > 2σ(*I*)
graphite	*R*_int_ = 0.031
Detector resolution: 15.9149 pixels mm^-1^	θ_max_ = 25.0°, θ_min_ = 3.6°
ω scans	*h* = −13→13
Absorption correction: multi-scan (*CrysAlis PRO*; Oxford Diffraction, 2009)	*k* = 0→6
*T*_min_ = 0.959, *T*_max_ = 0.969	*l* = 0→21
4288 measured reflections	

### Refinement

**Table:**

Refinement on *F*^2^	Primary atom site location: structure-invariant direct methods
Least-squares matrix: full	Secondary atom site location: difference Fourier map
*R*[*F*^2^ > 2σ(*F*^2^)] = 0.036	Hydrogen site location: inferred from neighbouring sites
*wR*(*F*^2^) = 0.067	H atoms treated by a mixture of independent and constrained refinement
*S* = 0.72	*w* = 1/[σ^2^(*F*_o_^2^) + (0.0266*P*)^2^] where *P* = (*F*_o_^2^ + 2*F*_c_^2^)/3
2136 reflections	(Δ/σ)_max_ = 0.002
186 parameters	Δρ_max_ = 0.11 e Å^−^^3^
0 restraints	Δρ_min_ = −0.13 e Å^−^^3^

### Special details

**Table:**

Experimental. Empirical absorption correction using spherical harmonics, implemented in SCALE3 ABSPACK scaling algorithm CrysAlisPro (Oxford Diffraction, 2009).
Geometry. Bond distances, angles etc. have been calculated using the rounded fractional coordinates. All su's are estimated from the variances of the (full) variance-covariance matrix. The cell esds are taken into account in the estimation of distances, angles and torsion angles
Refinement. Refinement of F^2^ against ALL reflections. The weighted R-factor wR and goodness of fit S are based on F^2^, conventional R-factors R are based on F, with F set to zero for negative F^2^. The threshold expression of F^2^ > 2sigma(F^2^) is used only for calculating R-factors(gt) etc. and is not relevant to the choice of reflections for refinement. R-factors based on F^2^ are statistically about twice as large as those based on F, and R- factors based on ALL data will be even larger.

### Fractional atomic coordinates and isotropic or equivalent isotropic displacement parameters (Å^2^)

**Table:**

	*x*	*y*	*z*	*U*_iso_*/*U*_eq_	
O1	0.59888 (11)	0.25607 (18)	0.48396 (8)	0.0491 (5)	
O2	0.40014 (13)	0.1526 (2)	0.68676 (8)	0.0710 (7)	
O3	0.80499 (13)	0.8397 (2)	0.49453 (8)	0.0645 (6)	
N1	0.67397 (13)	0.6177 (2)	0.57838 (9)	0.0420 (6)	
N2	0.59925 (13)	0.4517 (2)	0.59460 (9)	0.0425 (6)	
N3	0.48790 (15)	0.1552 (3)	0.57835 (10)	0.0454 (7)	
C1	0.79653 (19)	1.2321 (3)	0.80751 (14)	0.0665 (10)	
C2	0.8627 (2)	1.4184 (4)	0.79511 (16)	0.0705 (10)	
C3	0.90328 (19)	1.4454 (3)	0.72772 (16)	0.0626 (9)	
C4	0.87808 (17)	1.2884 (3)	0.66974 (14)	0.0504 (9)	
C5	0.80840 (16)	1.1006 (3)	0.68128 (12)	0.0434 (8)	
C6	0.77027 (17)	1.0762 (3)	0.75298 (13)	0.0549 (9)	
C7	0.91986 (18)	1.3147 (3)	0.59955 (15)	0.0606 (10)	
C8	0.89532 (17)	1.1653 (4)	0.54362 (13)	0.0589 (9)	
C9	0.82579 (17)	0.9788 (3)	0.55419 (13)	0.0495 (9)	
C10	0.78023 (16)	0.9449 (3)	0.62117 (12)	0.0413 (8)	
C11	0.70303 (15)	0.7611 (3)	0.63076 (11)	0.0436 (7)	
C12	0.56648 (16)	0.2867 (3)	0.54520 (12)	0.0398 (8)	
C13	0.46509 (18)	0.2336 (3)	0.64613 (12)	0.0503 (9)	
C14	0.53798 (18)	0.4406 (3)	0.66051 (11)	0.0496 (8)	
H1	0.76960	1.21310	0.85380	0.0800*	
H2	0.87910	1.52400	0.83260	0.0850*	
H3	0.94840	1.56920	0.71970	0.0750*	
H3N	0.4590 (16)	0.028 (3)	0.5590 (11)	0.065 (7)*	
H4	0.76770	0.73180	0.50630	0.0970*	
H6	0.72670	0.95210	0.76300	0.0660*	
H7	0.96540	1.43790	0.59150	0.0730*	
H8	0.92460	1.18630	0.49810	0.0710*	
H11	0.67310	0.74520	0.67630	0.0520*	
H14A	0.48920	0.57040	0.66440	0.0600*	
H14B	0.59220	0.42690	0.70580	0.0600*	

### Atomic displacement parameters (Å^2^)

**Table:**

	*U*^11^	*U*^22^	*U*^33^	*U*^12^	*U*^13^	*U*^23^
O1	0.0538 (10)	0.0529 (8)	0.0415 (10)	−0.0044 (7)	0.0094 (8)	−0.0084 (7)
O2	0.0901 (13)	0.0776 (10)	0.0488 (11)	−0.0257 (9)	0.0233 (9)	0.0057 (9)
O3	0.0725 (12)	0.0686 (10)	0.0544 (11)	−0.0094 (8)	0.0156 (9)	−0.0054 (9)
N1	0.0401 (10)	0.0373 (9)	0.0473 (12)	−0.0016 (8)	0.0000 (9)	−0.0017 (9)
N2	0.0467 (11)	0.0387 (10)	0.0424 (12)	−0.0071 (9)	0.0067 (9)	−0.0046 (9)
N3	0.0522 (12)	0.0420 (11)	0.0421 (13)	−0.0091 (9)	0.0061 (10)	−0.0013 (10)
C1	0.0649 (16)	0.0670 (16)	0.0662 (18)	−0.0015 (14)	0.0014 (14)	−0.0187 (14)
C2	0.0648 (18)	0.0583 (16)	0.083 (2)	0.0033 (13)	−0.0137 (16)	−0.0216 (15)
C3	0.0477 (15)	0.0391 (13)	0.095 (2)	−0.0026 (11)	−0.0162 (15)	−0.0068 (15)
C4	0.0383 (14)	0.0375 (13)	0.0718 (18)	0.0023 (11)	−0.0083 (12)	0.0001 (13)
C5	0.0343 (12)	0.0361 (12)	0.0576 (16)	0.0059 (10)	−0.0042 (11)	−0.0009 (11)
C6	0.0523 (15)	0.0508 (14)	0.0602 (17)	−0.0031 (11)	0.0014 (13)	−0.0107 (13)
C7	0.0455 (15)	0.0441 (14)	0.090 (2)	−0.0063 (11)	−0.0017 (15)	0.0105 (14)
C8	0.0478 (14)	0.0590 (15)	0.0704 (18)	−0.0027 (12)	0.0097 (13)	0.0143 (14)
C9	0.0432 (14)	0.0457 (13)	0.0586 (17)	0.0027 (11)	0.0013 (12)	−0.0004 (12)
C10	0.0328 (12)	0.0372 (12)	0.0532 (15)	−0.0012 (10)	0.0017 (11)	0.0029 (11)
C11	0.0457 (13)	0.0405 (11)	0.0441 (14)	0.0024 (11)	0.0037 (11)	−0.0020 (11)
C12	0.0377 (13)	0.0386 (13)	0.0417 (14)	0.0016 (10)	−0.0010 (11)	0.0013 (11)
C13	0.0601 (16)	0.0494 (14)	0.0406 (15)	−0.0026 (12)	0.0029 (12)	0.0028 (12)
C14	0.0585 (15)	0.0488 (13)	0.0410 (14)	−0.0022 (11)	0.0036 (12)	−0.0052 (10)

### Geometric parameters (Å, °)

**Table:**

O1—C12	1.219 (3)	C5—C10	1.440 (3)
O2—C13	1.209 (3)	C5—C6	1.419 (3)
O3—C9	1.361 (3)	C7—C8	1.355 (3)
O3—H4	0.8200	C8—C9	1.405 (3)
N1—N2	1.3726 (19)	C9—C10	1.385 (3)
N1—C11	1.293 (2)	C10—C11	1.443 (3)
N2—C12	1.357 (2)	C13—C14	1.508 (3)
N2—C14	1.451 (3)	C1—H1	0.9300
N3—C13	1.362 (3)	C2—H2	0.9300
N3—C12	1.389 (3)	C3—H3	0.9300
N3—H3N	0.890 (18)	C6—H6	0.9300
C1—C6	1.366 (3)	C7—H7	0.9300
C1—C2	1.389 (3)	C8—H8	0.9300
C2—C3	1.360 (4)	C11—H11	0.9300
C3—C4	1.413 (3)	C14—H14A	0.9700
C4—C7	1.413 (4)	C14—H14B	0.9700
C4—C5	1.416 (3)		
			
C9—O3—H4	109.00	N2—C12—N3	106.33 (17)
N2—N1—C11	116.51 (16)	O1—C12—N2	127.76 (17)
N1—N2—C14	125.77 (14)	O1—C12—N3	125.91 (18)
C12—N2—C14	112.17 (15)	O2—C13—N3	126.88 (18)
N1—N2—C12	121.80 (16)	O2—C13—C14	126.97 (19)
C12—N3—C13	113.02 (17)	N3—C13—C14	106.15 (17)
C13—N3—H3N	123.1 (13)	N2—C14—C13	102.24 (15)
C12—N3—H3N	123.7 (13)	C2—C1—H1	119.00
C2—C1—C6	121.3 (2)	C6—C1—H1	119.00
C1—C2—C3	119.5 (2)	C1—C2—H2	120.00
C2—C3—C4	121.1 (2)	C3—C2—H2	120.00
C3—C4—C5	119.8 (2)	C2—C3—H3	119.00
C3—C4—C7	121.57 (18)	C4—C3—H3	119.00
C5—C4—C7	118.64 (19)	C1—C6—H6	119.00
C4—C5—C10	119.44 (19)	C5—C6—H6	119.00
C4—C5—C6	117.23 (19)	C4—C7—H7	119.00
C6—C5—C10	123.33 (17)	C8—C7—H7	119.00
C1—C6—C5	121.07 (18)	C7—C8—H8	120.00
C4—C7—C8	121.78 (19)	C9—C8—H8	120.00
C7—C8—C9	120.2 (2)	N1—C11—H11	119.00
C8—C9—C10	121.05 (19)	C10—C11—H11	119.00
O3—C9—C10	123.12 (17)	N2—C14—H14A	111.00
O3—C9—C8	115.82 (19)	N2—C14—H14B	111.00
C5—C10—C9	118.88 (17)	C13—C14—H14A	111.00
C5—C10—C11	119.83 (18)	C13—C14—H14B	111.00
C9—C10—C11	121.27 (18)	H14A—C14—H14B	109.00
N1—C11—C10	122.37 (18)		
			
C11—N1—N2—C12	−177.23 (16)	C7—C4—C5—C10	−1.2 (3)
C11—N1—N2—C14	9.1 (2)	C3—C4—C7—C8	−179.6 (2)
N2—N1—C11—C10	−179.57 (16)	C5—C4—C7—C8	−0.2 (3)
N1—N2—C12—O1	2.8 (3)	C4—C5—C6—C1	2.3 (3)
N1—N2—C12—N3	−177.61 (15)	C10—C5—C6—C1	−178.07 (19)
C14—N2—C12—O1	177.26 (19)	C4—C5—C10—C9	2.3 (3)
C14—N2—C12—N3	−3.1 (2)	C4—C5—C10—C11	−175.93 (17)
N1—N2—C14—C13	176.87 (16)	C6—C5—C10—C9	−177.34 (18)
C12—N2—C14—C13	2.7 (2)	C6—C5—C10—C11	4.4 (3)
C13—N3—C12—O1	−178.02 (19)	C4—C7—C8—C9	0.6 (3)
C13—N3—C12—N2	2.4 (2)	C7—C8—C9—O3	179.31 (19)
C12—N3—C13—O2	179.8 (2)	C7—C8—C9—C10	0.6 (3)
C12—N3—C13—C14	−0.7 (2)	O3—C9—C10—C5	179.36 (17)
C6—C1—C2—C3	−0.7 (3)	O3—C9—C10—C11	−2.4 (3)
C2—C1—C6—C5	−0.9 (3)	C8—C9—C10—C5	−2.1 (3)
C1—C2—C3—C4	0.8 (3)	C8—C9—C10—C11	176.19 (18)
C2—C3—C4—C5	0.7 (3)	C5—C10—C11—N1	179.33 (17)
C2—C3—C4—C7	−179.9 (2)	C9—C10—C11—N1	1.1 (3)
C3—C4—C5—C6	−2.2 (3)	O2—C13—C14—N2	178.40 (19)
C3—C4—C5—C10	178.15 (18)	N3—C13—C14—N2	−1.1 (2)
C7—C4—C5—C6	178.47 (18)		

### Hydrogen-bond geometry (Å, °)

**Table:**

*D*—H···*A*	*D*—H	H···*A*	*D*···*A*	*D*—H···*A*
N3—H3N···O1^i^	0.890 (18)	1.962 (18)	2.851 (2)	177.9 (19)
O3—H4···N1	0.82	1.91	2.622 (2)	145
C6—H6···O2^ii^	0.93	2.55	3.469 (2)	169
C14—H14B···O2^ii^	0.97	2.36	3.038 (2)	127

Symmetry codes: (i) −*x*+1, −*y*, −*z*+1; (ii) −*x*+1, *y*+1/2, −*z*+3/2.
